# Platelet-rich concentrate in serum free medium enhances osteogenic differentiation of bone marrow-derived human mesenchymal stromal cells

**DOI:** 10.7717/peerj.2347

**Published:** 2016-09-07

**Authors:** Shani Samuel, Raja Elina Ahmad, Thamil Selvee Ramasamy, Puvanan Karunanithi, Sangeetha Vasudevaraj Naveen, Malliga Raman Murali, Azlina A. Abbas, Tunku Kamarul

**Affiliations:** 1 Department of Physiology, Faculty of Medicine, University of Malaya, Kuala Lumpur, Malaysia; 2 Department of Orthopedic Surgery, Faculty of Medicine, University of Malaya, Kuala Lumpur, Malaysia; 3 Department of Molecular Medicine, Faculty of Medicine, University of Malaya, Kuala Lumpur, Malaysia

**Keywords:** Stem cell, Blood, Orthopaedic, Growth factor, Regenerative medicine, Bone

## Abstract

Previous studies have shown that platelet concentrates used in conjunction with appropriate growth media enhance osteogenic differentiation of human mesenchymal stromal cells (hMSCs). However, their potential in inducing osteogenesis of hMSCs when cultured in serum free medium has not been explored. Furthermore, the resulting osteogenic molecular signatures of the hMSCs have not been compared to standard osteogenic medium. We studied the effect of infrequent supplementation (8-day interval) of 15% non-activated platelet-rich concentrate (PRC) in serum free medium on hMSCs proliferation and differentiation throughout a course of 24 days, and compared the effect with those cultured in a standard osteogenic medium (OM). Cell proliferation was analyzed by alamar blue assay. Gene expression of osteogenic markers (Runx2, Collagen1, Alkaline Phosphatase, Bone morphogenetic protein 2, Osteopontin, Osteocalcin, Osteonectin) were analyzed using Q-PCR. Immunocytochemical staining for osteocalcin, osteopontin and transcription factor Runx2 were done at 8, 16 and 24 days. Biochemical assays for the expression of ALP and osteocalcin were also performed at these time-points. Osteogenic differentiation was further confirmed qualitatively by Alizarin Red S staining that was quantified using cetylpyridinium chloride. Results showed that PRC supplemented in serum free medium enhanced hMSC proliferation, which peaked at day 16. The temporal pattern of gene expression of hMSCs under the influence of PRC was comparable to that of the osteogenic media, but at a greater extent at specific time points. Immunocytochemical staining revealed stronger staining for Runx2 in the PRC-treated group compared to OM, while the staining for Osteocalcin and Osteopontin were comparable in both groups. ALP activity and Osteocalcin/DNA level were higher in the PRC group. Cells in the PRC group had similar level of bone mineralization as those cultured in OM, as reflected by the intensity of Alizarin red stain. Collectively, these results demonstrate a great potential of PRC alone in inducing proliferation of hMSCs without any influence from other lineage-specific growth media. PRC alone has similar capacity to enhance hMSC osteogenic differentiation as a standard OM, without changing the temporal profile of the differentiation process. Thus, PRC could be used as a substitute medium to provide sufficient pool of pre-differentiated hMSCs for potential clinical application in bone regeneration.

## Introduction

Non-union of the bone pose a major problem in the field of orthopedics, necessitating the need to explore more effective treatment strategies (Dimitriou et al., 2011, Grayson et al., 2015). Novel strategies currently used to facilitate bone regeneration include the use of scaffolds, growth factors, cells, or a combination of the three. Although these strategies may generally produce satisfactory clinical outcomes, none have demonstrated the ability to mimic the normal cascade of bone formation. Hence, efforts aimed at exploring the interactions between osteogenic cell populations from divergent sources and various osteoinductive stimuli have been a major focus of current research (Giannoudis, Einhorn & Marsh, 2007).

The use of mesenchymal stromal cells (MSCs) as a source for bone repair has been greatly considered in view of their remarkable ability to expand extensively and differentiate into multiple cell types *in vitro* under appropriate physiological conditions. It has been demonstrated that signaling factors, such as growth/ differentiation factor supplemented in the culture media provide favorable conditions for cells to thrive. Recent studies have shown that transplanting osteogenic pre-differentiated cells accelerate the repair of bone injury by enhancing the formation of collagen type I-rich matrix (Clough et al., 2015; Han et al., 2015; Ye et al., 2012). Utilization of pre-differentiated allogeneic MSCs could circumvent the limitations associated with the use of autologous cells. These include issues such as limited availability of tissues for cell isolation, chronic pain during tissue harvest, and morbidity at the donor site (Anderson et al., 2014). Accordingly, much effort has been directed to investigate various ways to augment the differentiation potential of MSCs *in vitro*; one of which is exploring the potential application of naturally occurring autologous growth factor reservoirs such as platelet-rich plasma (PRP). The release of growth factors from the platelets appears to aid in various aspects of tissue regeneration such as chemotaxis, proliferation, differentiation, and angiogenesis (Argintar, Edwards & Delahay, 2011; Durante et al., 2013; Eppley, Pietrzak & Blanton, 2006).

Previous studies have investigated the effect of PRP on the proliferation and differentiation potential of MSCs, particularly towards osteogenic lineage. However, the results have remained inconclusive. Few studies have indicated that PRP supports osteogenic differentiation of MSC (Parsons et al., 2008; Van den Dolder et al., 2006; Verrier et al., 2010), while others demonstrated an inhibitory effect (Gruber et al., 2006; Lee et al., 2014b). Most studies used platelet concentrates only to expand the MSC population. These cells were then cultured in specific differentiation media to induce lineage-specific differentiation (Iudicone et al., 2014; Li et al., 2013). Although the effect of platelet rich concentrate (PRC) in augmenting MSC proliferation is well established, studies reporting its role as an isolated medium to induce osteogenic differentiation of MSCs *in vitro* appear to be limited. In addition, since the effect of platelet concentrates alone has not been directly compared to osteogenic medium in a single experimental setting, it remains unclear whether PRC would confer added benefits than the commercially available osteogenic inductive medium in promoting the differentiation of MSCs *in vitro*. Hence, in this study, we sought to clarify the following issues: (1) whether PRC alone can be used as an isolated growth medium to induce osteogenic differentiation of hMSCs, (2) whether the molecular signature of osteogenic markers induced by PRC are comparable to that induced by commercially available osteogenic medium, and (3) whether any deduction could be made based on the observed molecular signature as to whether PRC modulates key event(s) in the osteogenic differentiation process of hMSCs. This would provide a greater insight into understanding the effect of PRC in serum free medium on the differentiation potential of hMSCs towards the osteogenic lineage. This study has an implication of potentially advocating the use of PRC as an osteoinductive medium to substitute the commercially available growth medium to obtain sufficient pre-differentiated MSCs for *in vivo* bone regeneration applications.

## Materials and Methods

### Human mesenchymal stromal cell (hMSC) isolation

Bone marrow was aspirated from patients undergoing total knee/hip arthroplasty in the University of Malaya Medical Centre. The study was approved by the Medical Ethics Committee of the institution (UMMC, reference number 967.10). Written informed consent was obtained from each participant. Aspirated bone marrow was added to equal volume of phosphate-buffered saline (PBS; pH 7.2; Invitrogen-Gibco, MA, USA) and layered onto Ficoll-Paque Premium of density 1.073 g/mL (GE Healthcare, Uppsala, Sweden). The samples were then centrifuged at 360× g for 25 min. The mononuclear cells were later isolated and resuspended in 10 mL of low glucose Dulbecco’s modified eagle medium (L-DMEM) (Invitrogen-Gibco) and centrifuged again at 220× g for 5 min. The supernatant was discarded and the cell pellet obtained was cultured in growth medium (L-DMEM supplemented with 10% fetal bovine serum (FBS), 1% Penicillin/Streptomycin (100 U/mL; Invitrogen-Gibco) and 1% Glutamax-1 (Invitrogen-Gibco) in T-25 tissue culture flasks. Medium was changed every three days until the cultures were 80% confluent. The cells were then serially passaged until passage 2, which were harvested and used for further experiments.

Before proceeding with the experiment, we performed additional experimental procedures to verify that the isolated cells were mesenchymal stromal cells, based on their ability to differentiate to adipocyte, osteoblast and chondrocyte. The experiments were carried out as described earlier (Nam et al., 2013). The formation of lipid droplets and mineralization indicated the potential of cells to differentiate to the adipogenic and osteogenic lineage, respectively. The ability of the cells to differentiate to chondrocytes was reflected by the intensity of the red staining for proteoglycans deposited in the matrix. Our results confirmed that the isolated cells were mesenchymal stromal cells (data not shown).

### Preparation and addition of PRC

PRC was prepared from a pool of blood from six healthy volunteers. Blood samples for PRC preparation and bone marrow harvested for hMSCs isolation originated from different group of donors. Twenty-five milliliters of blood from each subject was collected in ACD tubes. Platelets were prepared using double centrifugation method as mentioned previously, with slight modifications (Cho et al., 2011; Lee et al., 2012; Shani et al., 2014). In brief, the first centrifugation was at 200× g for 10 min, which separated the red blood cells, plasma and buffy coat that contains the platelets and the white blood cells. The plasma and buffy coat obtained were pooled in a 50 mL falcon tube in order to minimize inter-individual variability. Prostacyclin (PGI_2_) was added to prevent the transitory activation of platelets during the centrifugation and re-suspension steps. The pooled plasma was then centrifuged at 800× g for another 10 min. The supernatant portion of the plasma was discarded and only the platelet pellets were isolated and resuspended in sterile phosphate buffered saline (PBS, pH 7.2), at 1/10th the initial blood volume. This constitutes the platelet-rich concentrate. Concentration of major platelet growth factors i.e., PDGF-AA, PDGF-AB, PDGF-BB and TGF-*β*1 in PRC was determined using ELISA (USCN, Schilde, Belgium) according to the manufacturer’s instructions to verify that the medium qualified as PRC. In our preparation, the concentration of PDGF-AA, PDGF-AB, PDGF-BB and TGF-*β*1 was found to be 50.32 ng/mL, 9.91 ng/mL, 8.32 ng/mL and 15.17 ng/mL respectively. These results were previously reported elsewhere (Shani et al., 2014).

### *In vitro* cell viability assay

MSC viability assay was performed to verify that PRC alone could expand and sustain cell viability. Human MSCs were seeded in 24-well culture plates at a density of 1.5 × 10^3^ cells/well. After 48 hours of culture in growth medium, the cells were subjected to serum reduction of 1% FBS to arrest cell cycle progression for 24 hours. The cells were then either supplemented with normal growth medium containing L-DMEM supplemented with 10% FBS, 1% Penicillin/Streptomycin and 1% Glutamax-1 (all from Invitrogen-Gibco), which served as the control, or, the FBS was replaced with PRC. Our previous study indicated that 15% PRC resulted in optimal cell viability (Shani et al., 2014), and hence, the serum free medium was supplemented with 15% PRC in the experimental wells. Media was changed every 8 days and fresh PRC was also supplemented every 8 days. Cell viability was observed at 0, 8, 16 and 24 days using alamarBlue^®^ assay (Life Technologies, CA, USA) according to the manufacturer’s protocol. All experiments were performed in triplicate and repeated three times.

### *In vitro* cell differentiation

Approximately 800 cells/cm^2^ were cultured in 24-well plates and the media were either supplemented with 10% FBS (negative control) or osteogenic medium (StemPro^®^, Invitrogen) (positive control) or 15% PRC. The commercially available osteogenic medium is serum free but contains dexamethasone, ascorbic acid and growth factors known to induce osteogenic differentiation. In this study, 10% FBS was used as the negative control as the supplements in FBS aid only in maintaining the cell viability and proliferation, but they do not significantly induce cell differentiation.

### Quantitative real-time PCR

Quantitative real-time reverse transcription polymerase chain reaction (qRT-PCR) analysis was performed to compare the relative expression of osteogenic genes. After 0, 8, 16 and 24 days, cells were detached using TypLE™(Invitrogen) express and cell pellets were lysed with buffer RLT (Qiagen, CA, USA). Total RNA was then isolated from the cells using RNeasy Mini Kit (Qiagen) in accordance with the manufacturer’s recommendations, and quantified with a nanophotometer (Implen GmbH, Germany). One µg of RNA was used to generate cDNA using QuantiTect Reverse Transcription kit (Qiagen) in accordance with the manufacturer’s instructions. Real-time PCR analysis (CFX96 Real-time system, Bio-Rad) was performed to assess the mRNA levels using QuantiTect SYBR^®^ Green PCR Kits (Qiagen). Each Q-PCR was performed four times for PCR yield validation. Data were analyzed by the 2^−ΔΔCt^ method, with normalization by the Ct of the housekeeping gene glyceraldehyde-3-phosphate dehydrogenase (GAPDH). Results were expressed as relative fold changes to the gene expression levels of hMSC cultured in FBS medium.

The primers used for Q-PCR are summarized in Table 1.

**Table 1:** List of primers for Q-PCR.

Gene	Primer sequences	Length (Base pairs)
Collagen 1F	CCCGCAGGCTCCTCCCAG	18
Collagen 1R	AAGCCCGGATCTGCCCTATTTAT	23
Osteopontin F	GTCCCCACAGTAGACACATATG	21
Osteopontin R	TCAACTCCTCGCTTTCCATG	20
Osteocalcin F	GGAGGGCAGCGAGGTAGTGAAGA	23
Osteocalcin R	GCCTCCTGAAAGCCGATGTGGT	22
RUNX2 F	CCGCCATGCACCACCACCT	19
RUNX2 R	CTGGGCCACTGCTGAGGAATTT	22
BMP2 F	TGGCCCACTTGGAGGAGAAACA	22
BMP2 R	CGCTGTTTGTGTTTGGCTTGACG	22
ALP F	GATGTGGAGTATGAGAGTGACG	22
ALP R	GGTCAAGGGTCAGGAGTTC	19
Osteonectin F	TTGCAATGGGCCACATACCT	20
Osteonectin R	GGGCCAATCTCTCCTACTGC	20
GAPDH F	GCCCCCTCTGCTGATGCCC	19
GAPDH R	GGGTGGCAGTGATGGCATGGA	21

### Immunocytochemistry

Immunocytochemistry for osteogenic markers Runx2, osteocalcin and osteopontin were performed at days 8, 16 and 24. Briefly, MSCs were fixed in 4% paraformaldehyde for 15 min, and was then blocked for 30 min using hydrogen peroxidase (H_2_O_2_) to prevent endogenous activity. Cells were then incubated in goat serum working solution for 15 min to block non-specific binding followed by the primary antibody (mouse anti-human monoclonal antibody, 1:100 dilution, Abcam, Cambridge, UK) at room temperature for 30 min. After washing with PBS, cells were incubated with goat anti-mouse secondary antibody tagged with Alexa fluoro (Abcam) (1:200 dilution) for 30 min. Cells were then washed with PBS and the nucleus was counterstained with Hoechst stain (NucBlue^®^ Live ReadyProbes^®^ Reagent; Life technologies) and incubated for 15 min. After washing with PBS, it was examined under the fluorescent microscope (Nikon Eclipse TE2000-S; Nikon, Tokyo, Japan).

### ALP and osteocalcin assay

For the biochemical assays, the cells were lysed with RIPA buffer. The samples were then centrifuged at 10,000× g for 5 min, and the clear supernatants were used (Gao et al., 2014). Cellular alkaline phosphates enzyme (ALP) activity, an early marker of osteogenic differentiation, was assessed at days 8, 16 and 24. ALP activity was determined colorimetrically by incubating the cell lysates with the substrate *p*NPP (*p*-nitrophenyl phosphate) (BioVision, CA, USA) in a 96-well plate at 37 °C for 60 min. ALP assay involves the dephosphorylation of *p*NPP by ALP to a yellow coloured *p*NP (*p*-nitrophenol), the absorbance of which was measured at 405 nm. The enzyme activity was normalized with total DNA content and expressed as nmol/ng.

Quantification of osteocalcin in cell lysates was performed at days 8, 16 and 24 and in accordance with the manufacturer’s instructions using the Gla-type Osteocalcin EIA kit (TAKARA, Shiga, Japan). The absorbance was measured at 450 nm in duplicates. The amount of osteocalcin was determined by interpolating the values from a standard curve of known concentrations. The enzyme activity was normalized with total DNA content and expressed as ng/ng. Total DNA was assessed in digested samples using a Quant-iT PicoGreen dsDNA kit according to manufacturer’s instructions (Molecular Probes, Invitrogen).

### Alizarin red staining and quantification

A quantitative Alizarin Red S method was used at 8, 16 and 24 days to detect calcium compounds deposited in the extracellular matrix (ECM) as a result of bone mineralization. The cells were later fixed in 4% paraformaldehyde for 20 min. The fixed cultures were stained with 2% Alizarin Red S (Sigma-Aldrich), pH 4.2 for 10 min. Stained monolayers were visualized under the microscope (Nikon Eclipse). Quantification of the stain was performed with cetylpyridinium chloride (CPC) (Sigma-Aldrich). Briefly, 10% (w/v) cetylpyridinium chloride was prepared in PBS (pH 7.0). Stained monolayers were incubated with 1 mL of CPC with shaking for 15 min. 200 µL aliquots of the extracted dye was then transferred to 96 well plates and absorbance was measured at 570 nm (Frazier et al., 2013; Lee et al., 2014a).

### Statistical analysis

Values are expressed as mean ± standard deviation. The differences between groups were analyzed using a non-parametric test (Kruskal–Wallis). If values were significant, Mann Whitney U tests were performed to determine the level of significance between the groups. Differences were considered to be significant at *p* ≤ 0.05. Data were analyzed using SPSS software version 17.0 (IBM Corp., Armonk, NY, USA).

## Results

### Platelet yield and its effect on proliferation of hMSC

**Figure 1:**
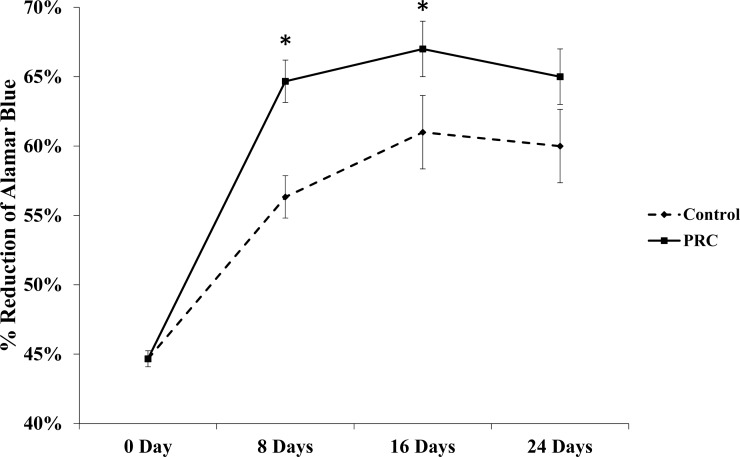
Effect of PRC on hMSC proliferation. Cells treated with PRC had a significant increase in proliferation compared to the control at day 8 and 16. Data are represented as the means ± SD. * denotes statistical significance (*p* < 0.05).

Our PRC preparation yielded approximately four fold higher platelet concentration (1157 ± 92.37 × 10^3^ platelets/µL) than that in the whole blood (263.71 ± 22.83 × 10^3^ platelets/µL) (*p* = 0.021). Our results also showed that PRC supplemented in serum free medium significantly increased the cell number by day 8 and maintained the cell viability for the remaining period of study, as shown in Fig. 1.

### Gene expression of osteogenic markers in hMSC cultured in PRC-supplemented media

**Figure 2:**
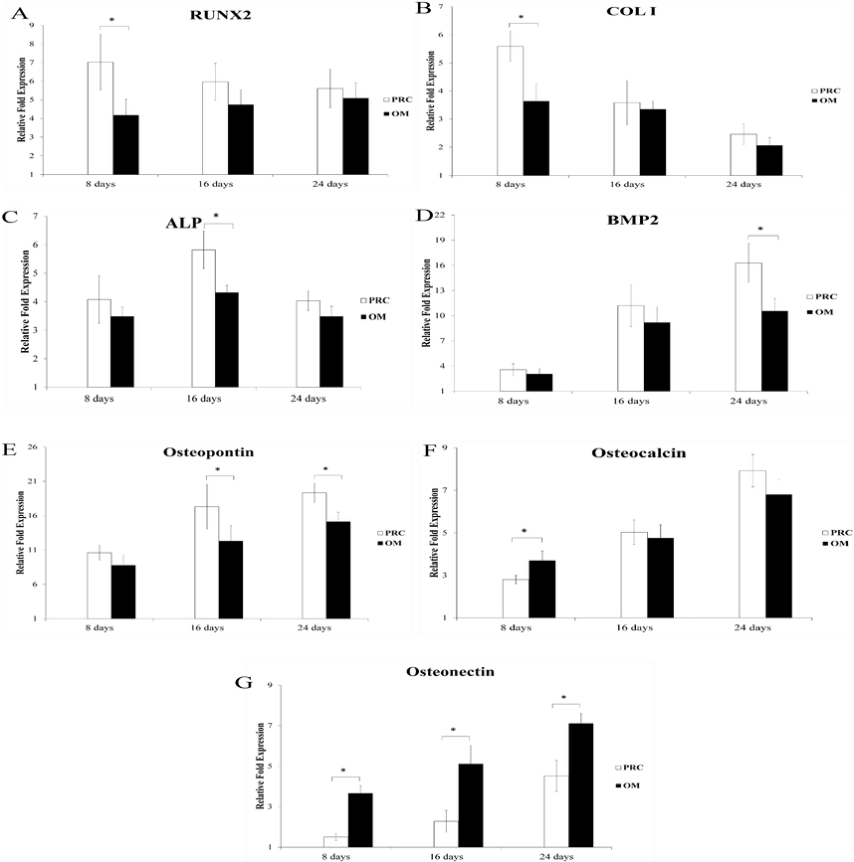
Expression of osteogenic genes throughout the experiment. PRC significantly increased the expression of Runx2 (A) , Col I (B), ALP (C), BMP2 (D) and osteopontin (E) compared to the osteogenic medium (OM) at various specific time points. No significant difference was observed in the expression of osteocalcin (F) by day 24. On the contrary, osteonectin (G) expression was significantly lower in the PRC group compared to the OM throughout the course of study. Results are expressed as fold change relative to the control (FBS medium). Data are expressed as the mean ± SD. * denotes statistical significance between the indicated pairs (*p* < 0.05).

The temporal pattern of expression of osteogenic gene markers in the PRC group was similar to that in the osteogenic medium throughout the duration of culture; however, the expression of certain osteogenic genes were significantly higher in the PRC group compared to the osteogenic medium at specific time points (Fig. 2). At the early time point (day 8), PRC upregulated the expression of transcription factor Runx2 by 3-fold and collagen type I (Col 1) by 2-fold compared to the osteogenic medium. The expression of ALP was 1-fold higher in the PRC group compared to the osteogenic medium (*p* < 0.05) at day 16. By day 24, expression of bone morphogenetic protein 2 (BMP2), one of the key factors in osteogenesis, increased by approximately 6-fold in the PRC group compared to the osteogenic medium (*p* < 0.05). The expression of another ECM marker, osteopontin, produced by the osteoblasts was also significantly higher by 5-fold at day 16 and 4-fold at day 24 in the PRC group compared to the osteogenic medium. On the other hand, throughout the duration of cell culture, the expression of osteonectin, a late osteogenic marker, seemed to be persistently upregulated in the OM compared to the PRC supplement (*p* < 0.05).

### Immunocytochemical staining of osteogenic markers

**Figure 3:**
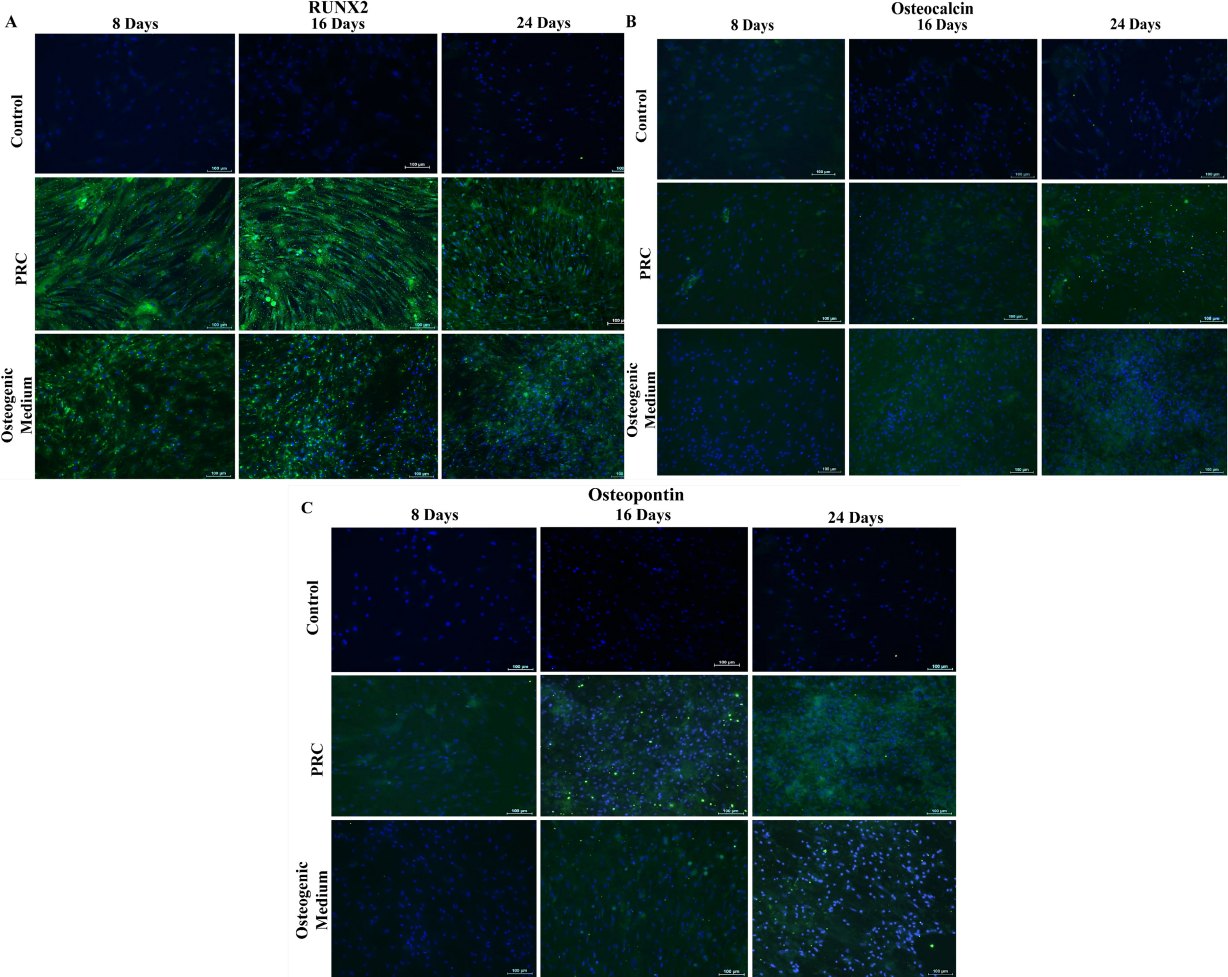
Immunocytochemical staining for osteogenic differentiation markers. Cells treated with PRC had stronger positive staining for Runx2 compared to the cells cultured in osteogenic medium, which was observed by day 8. Both osteocalcin and osteopontin were detected at a comparable level to the osteogenic medium. Expression of the proteins in the cytoplasm was reflected by green fluorescence and nucleus was stained blue by Hoechst. The above are representative images viewed at 10× magnification (scale bar: 100 μM).

Immunocytochemical analysis showed that in comparison with cells cultured in the osteogenic medium, cells treated with PRC had stronger staining for Runx2 (Fig. 3A) and comparable staining intensity for both Osteocalcin (Fig. 3B) and Osteopontin (Fig. 3C). Staining for all three markers were absent in cells cultured in the FBS-containing medium.

### ALP and osteocalcin assays

**Figure 4:**
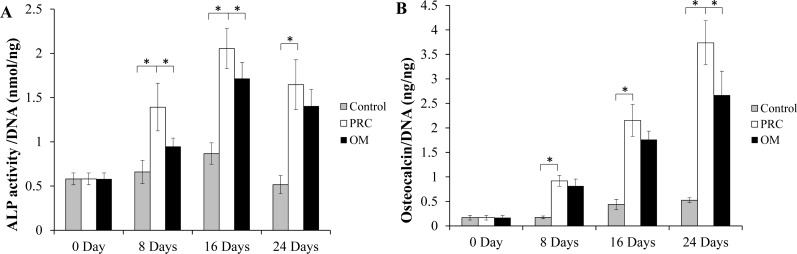
ALP activity and Osteocalcin protein quantification. (A) ALP activity was significantly higher in the PRC treated cells compared to other groups at day 8 and 16; (B) Osteocalcin level gradually increased, peaking at day 24, with PRC group having the highest concentration throughout the study duration. The ALP activity and osteocalcin levels in each sample were normalized to the amount of DNA in the sample. Data are represented as the means ± SD. * denotes statistical significance between the indicated pairs (*p* < 0.05). (OM denotes osteogenic medium group).

There was a significant increase in ALP activity of cells in both the PRC and osteogenic medium (*p* < 0.05) compared to the cells in the FBS medium (Fig. 4A). The level appeared to be slightly higher in the PRC group compared to that in the osteogenic medium at day 8 and 16 (*p* < 0.05). There was a gradual increment of osteocalcin protein expression throughout the duration of cell culture. Similar to the ALP activity, osteocalcin level was also significantly higher in cells cultured in PRC and osteogenic medium compared to the control group (*p* < 0.05) (Fig. 4B). However, cells in the PRC group had a higher level of osteocalcin compared to the osteogenic medium (*p* < 0.05).

### Alizarin red staining and quantification

**Figure 5:**
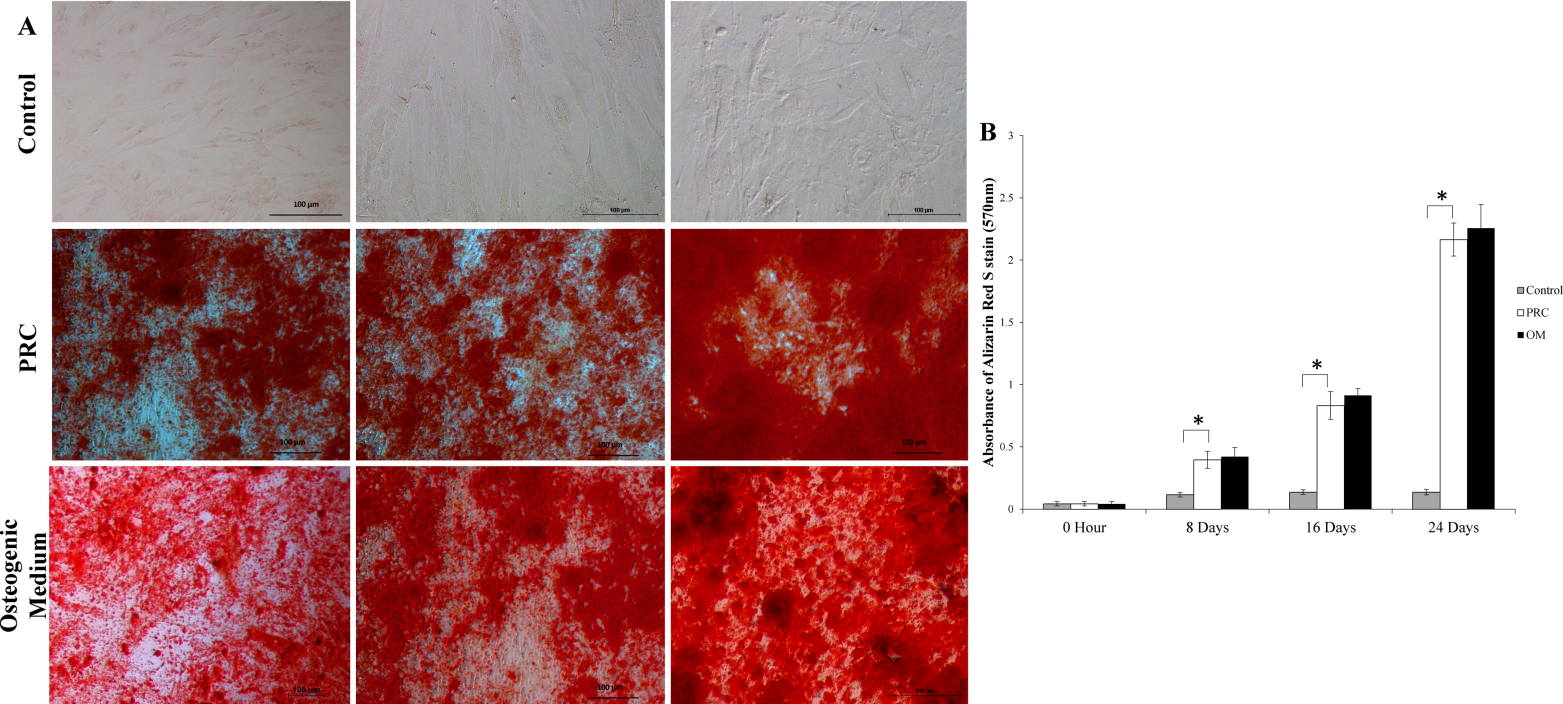
Mineralization detected by alizarin red *S* staining and Quantification. (A) The differentiated cells showed strong Alizarin red S staining. (B) The absorbance value of solubilized alizarin red was high in the PRC-treated cells at all-time points compared to the control but comparable to the cells in OM. Data are represented as the means ± SD. * denotes statistical significance between the indicated pairs (*p* < 0.05).

The mineralized large nodules formed by cells cultured in PRC were intensely stained with Alizarin red S stain, indicating a higher degree of mineralization compared to the control group (Fig. 5A). Throughout the course of the study, there was a gradual increase in the absorbance of the Alizarin Red S stain (quantified by the extraction of the calcified mineral from the stained monolayer) (Fig. 5B), which is indicative of bone mineralization in the differentiated cells. PRC group had significantly higher bone mineralization compared to the control group. Its level was comparable to that of the osteogenic medium, indicating that PRC was equally effective in inducing mineralization as the osteogenic medium.

## Discussion

The present study investigated the effect of PRC as an isolated medium to induce osteogenic differentiation of hMSCs when compared to the commercially available osteogenic medium. This effect was reflected by the changes in the molecular signatures of hMSCs cultured in both media. Our results showed that infrequent supplementation of PRC alone enhances osteogenic differentiation of hMSCs. Unlike previous studies that exercised frequent PRC supplementation (e.g., every 3 days) in combination with foetal bovine serum (FBS), we have taken the approach to supplement only PRC at an 8-day interval. This allows us to determine the efficacy of PRC alone in inducing hMSC proliferation and osteogenic differentiation without any confounding effect of other growth factors in the FBS. In addition, supplementing PRC at a longer interval also allows us to establish whether PRC could sustain osteogenic differentiation despite the declining supply of nutrition over time. Given the relatively short half-life of growth factors, it is reasonable to expect that their effect on the cells would rapidly decline after the first exposure thus, requiring more frequent addition of fresh platelets to sustain the desired outcome, as previously done in other studies (Lee et al., 2014b; Verrier et al., 2010). The enhancement of osteogenic differentiation of hMSCs under the influence of PRC alone, as observed in this study, implies that the growth factors in PRC were very potent as an osteogenic promoter. It also suggests that there might have been a gradual activation of the platelets resulting in slow release of the growth factors over a period of eight days, which was able to sustain the proliferative and osteoinductive effect of the PRC. It is plausible that a higher metabolic activity of the actively proliferating cells increases ATP hydrolysis, thus generating ADP molecules, which is a known potent platelet activator (Puri & Colman, 1997). Consistent with the findings of others, our results also showed that PRC induced greater proliferation of the hMSCs compared to FBS (Gruber et al., 2004; Lucarelli et al., 2003; Mishra et al., 2009). Various growth factors found in PRC such as platelet derived growth factor (PDGF), transforming growth factor (TGF), fibroblast growth factor (FGF) and epidermal growth factor (EGF) have been known to induce mitosis (Bertrand-Duchesne, Grenier & Gagnon, 2010; Lucarelli et al., 2003); which might explain the observations of our study.

Although the proliferative effect of PRC on hMSCs is well established, studies that investigate the osteogenic molecular signatures of hMSC under the influence of PRC alone appear to be lacking. Such information would be valuable in providing an insight into the regulation and molecular mechanism of osteogenesis, which may have been modulated by the growth factors in PRC. The present study provides a very comprehensive molecular signature of hMSCs during their osteogenic differentiation under the influence of both PRC and standard osteogenic media. The results indicate that although the temporal pattern of gene expression between the PRC group and the osteogenic medium group was similar, the magnitude of expression of most osteogenic markers was greater in the presence of PRC. As cells cultured in both media showed similar temporal profile of gene expressions, it can be speculated that the signaling pathways triggered in the PRC-treated cells may have been unaltered. However, using PRC to induce osteogenesis would appear to be more beneficial for clinical applications as it is an autologous source of growth factors, and hence, is free from xenogeneic components. Consequently, the use of PRC poses no risk of disease transmission or immunogenic reaction (Li et al., 2013). In this study, we used pooled platelets, which in our view, is acceptable for *in vitro* applications. Pooled allogeneic platelets might be beneficial for *in vivo* applications after undergoing further processing, which include pathogen inactivation (Iudicone et al., 2014) or irradiation (Fekete et al., 2012) to prevent possible disease transmission from human derived products. However, this warrants further investigation.

The ability of hMSCs to undergo osteogenic differentiation in the presence of PRC might be due to the presence of various growth factors, many of which have been recognized to have the ability to induce osteogenic differentiation of MSCs (Whiteheart, 2011). For example, bone morphogenetic protein (BMP2) and platelet derived growth factor (PDGF) are known to affect bone formation. BMP2 plays a key role in the osteoblast commitment and induce MSCs to form osteoblast by increasing the ALP activity, and thus plays a role in the early stage of osteogenic differentiation (Hughes et al., 1995). PDGF also regulates osteogenic differentiation by modulating BMP signaling (Li et al., 2014; Lysdahl et al., 2014; Zdunek et al., 2008). Based on our results, it can be speculated that the combination of these growth factors in PRC synergistically enhanced the differentiation potential of hMSCs towards osteogenic lineage. These growth factors trigger various signaling pathways, which eventually lead to the expression of the osteogenic markers. Among these pathways, Runx2 is regarded as the master regulator of osteogenesis, which is activated by BMP2 signaling cascade. Activation of BMP signaling during osteogenesis through Smad1/5/8 and MAPK downstream signaling stimulates Runx2 expression (James, 2013; Lee et al., 2000), which in turn induce the expression of osteoblastic markers osteocalcin, type I collagen and osteopontin (Jang et al., 2012; Welch et al., 1998). This speculation, however, needs to be investigated further, and an interrogation of the role of specific growth factors in PRC can be a focus of future research to further understand the mechanism underlying the valuable action of PRC in promoting osteogenic differentiation of hMSCs. Nevertheless, the present study is valuable in determining the merit of using PRC alone to differentiate hMSCs *in vitro* to provide sufficient pre-differentiated cells, which can then be used for enhancement of bone repair *in vivo*.

## Conclusion

Our results indicate that non-activated PRC alone, albeit infrequently supplemented, produces similar osteogenic molecular signature of hMSCs as compared to the commercially available osteogenic media. Thus, the supplementation of PRC in serum free medium could be an advantageous strategy for achieving hMSC differentiation *in vitro,* which may have the potential for clinical applications in conditions such as non-unions to augment bone regeneration *in vivo*.

##  Supplemental Information

10.7717/peerj.2347/supp-1 Data S1 Raw data for gene expressions

10.7717/peerj.2347/supp-2 Data S2 Raw data for cell proliferation

10.7717/peerj.2347/supp-3 Data S3 Raw data for ALP assay

10.7717/peerj.2347/supp-4 Data S4 Raw data for osteocalcin assay

10.7717/peerj.2347/supp-5 Data S5 Raw data for alizarin red absorbance

## Additional Information and Declarations

### Data Availability

The following information was supplied regarding data availability: The raw data has been supplied as Supplementary Files.
